# The Correlation between *Chlamydia Trachomatis* and Female Infertility: A Systematic Review

**DOI:** 10.1055/s-0042-1748023

**Published:** 2022-05-16

**Authors:** Laura Gazal Passos, Paula Terraciano, Nicole Wolf, Fernanda dos Santos de Oliveira, Isabel de Almeida, Eduardo Pandolfi Passos

**Affiliations:** 1 Medicine school of Pontifícia Universidade Católica do Rio Grande do Sul, Porto Alegre, RS, Brazil; 2 Center for Experimental Research, Hospital de Clínicas de Porto Alegre, Porto Alegre, Brazil; 3 Medicine school of Universidade Federal do Rio Grande do Sul, Porto Alegre, RS, Brazil; 4 Fertility Center, Hospital Moinhos de Vento, Porto Alegre, RS, Brazil

**Keywords:** chlamydia trachomatis, infertility, tubal factor infertility, sexually transmitted diseases, human reproduction, clamídia trachomatis, infertilidade, infertilidade tubária, infecções sexualmente transmissíveis, reprodução humana

## Abstract

The impact of *Chlamydia trachomatis* (CT) infection on female's fertility is not completely established yet, since the level of evidence associating these factors is still weak. Hence, the goal of the present review is to contribute to a better elucidation of this matter. The electronic database chosen was the Medline/PubMed, with the last survey on May 11, 2021. Publication date was used as a filter, with the previous 5 years having been selected. The following describers were used: *chlamydia trachomatis* AND *infertility* ; *chlamydia trachomatis* AND *tubal alteration* AND *infertility* ; *chlamydia* AND low *pregnancy rates* . From the 322 studies screened, 293 that failed to meet our eligibility criteria were excluded. Subsequently, we removed seven studies for not having the possible correlation between CT infections and female infertility as its main focus, and three for being about sexually transmitted infections (STIs) in general. Moreover, two studies designed as reviews were also excluded. Ergo, we included 17 studies in our qualitative analysis. The authors conducted research individually and analyzed carefully the studies selected. As we retrieved the information needed for our study through reading the texts, no contact was made with the authors of the studies selected. This systematic review corroborates the hypothesis that CT infection potentiates female infertility, as 76.47% of the included studies found a positive correlation between them. We conclude that there is an important association between CT infection and female infertility. Ergo, making CT screening part of the infertility investigation routine is relevant and has a reasonable justification.

## Resumo

O impacto da infecção por *Chlamydia trachomatis* (CT) na fertilidade feminina ainda não está completamente estabelecido, uma vez que o nível de evidência associando esses fatores ainda é insignificante. Assim, o objetivo desta revisão é contribuir para uma melhor elucidação deste assunto. A base de dados eletrônica escolhida foi a Medline/PubMed, com a última pesquisa em 11 de maio de 2021. Utilizou-se como filtro a data de publicação, sendo selecionados os 5 anos anteriores. Foram usados os seguintes descritores: *Chlamydia* *trachomatis* E *infertility* ; *Chlamydia* *trachomatis* E *tubal alteration* E *infertility* ; *Chlamydia* E *low pregnancy rates* . Dos 322 estudos selecionados, 293 que não atenderam aos nossos critérios de elegibilidade foram excluídos. Posteriormente, retiramos sete estudos por não terem como foco principal a possível correlação entre infecção por CT e infertilidade feminina e três por tratarem de infecções sexualmente transmissíveis (ISTs) em geral. Além disso, dois estudos concebidos como revisões também foram excluídos. Portanto, incluímos 17 estudos em nossa análise qualitativa. Os autores realizaram pesquisas individualmente e analisaram criteriosamente os estudos selecionados. Como obtivemos as informações necessárias para nosso estudo por meio da leitura dos textos, nenhum contato foi feito com os autores. Esta revisão sistemática corrobora a hipótese de que a infecção por CT potencializa a infertilidade feminina, pois 76,47% dos estudos incluídos encontraram correlação positiva entre eles. Concluímos que existe uma associação importante entre infecção por CT e infertilidade feminina. Portanto, tornar os procedimentos de triagem por CT parte da rotina de investigação de infertilidade é relevante e justificável.

## Introduction

*Chlamydia trachomatis* (CT) infections represents, globally, the most prevalent sexually transmitted infection (STI) caused by bacteria, with 131 million new cases per year. 1 2 *Chlamydia trachomatis,* which is an obligate intracellular parasite, can have a specific infectious potential to epithelial cells from male and female reproductive tracts. In symptomatic cases, men can present with urethritis, or, less commonly, epididymitis, and women, besides yellowish vaginal discharge, spontaneous bleeding, pain during sex or urination, and pelvic pain, may be led to pelvic inflammatory disease (PID). 3 4 However, most women and 50% of men affected do not present many identifiable clinical symptoms, having an unnoticed infection. 5 Therefore, the majority of infected individuals do not seek treatment, not only risking their sexual partners' health, but also worsening their condition, as the persistent presence of the pathogen evocates a chronic immune response, leading to an enhanced production of genital immune mediators, like interleukin (IL)-, IL-6 and gamma interferon, which increases the number of epithelial cells destroyed. 5 6 This process is very dangerous, especially among women, once the manifestations and consequences are more damaging to their reproductive health than man's, a fact elucidated by the evidence that approximately 20% of women with chlamydial lower genital tract infection will develop PID, 4% develop chronic pelvic pain, 2% adverse pregnancy outcomes (chromosomal abnormalities, miscarriages, congenital malformations and stillbirth) and 3% infertility—probably due to scar formation and occlusion of the Fallopian tubes. 7 The last possible consequence mentioned is defined as a couple's ineptitude to conceive after at least 12 months of regular unprotected intercourse and affects up to 15% of the reproductive-aged population. 8 Even though this issue is widely recognized amidst the medical community as a secondary effect of female CT infection, the level of evidence corroborating the association is relatively weak. 7 Thus, by doing a systematic review, we aim to contribute to the consolidation of this correlation.

## Methods

The development of this study was based on the review writing methods of the Preferred Reporting Items for Systematic reviews and Meta-Analyses (PRISMA) statement. 9

We included cohort studies, case-control studies, crosssectional studies, and a letter, which were performed in the last 5 years and contained data about the correlation between infertility among reproductive-aged women and previous CT infection. Additionally, studies that compared the fertility rates between women with and without positive immunoglobulin G (IgG) for CT were admitted in this systematic review. There was no language restriction for the studies selection. Articles related to endocrine causes or just male infertility were excluded, as well as studies focused on STI in general or on different from CT. Additionally, we ruled out articles perceived as duplicates and reviews about the subject.

The electronic database chosen to carry out the search was the Medline/PubMed, with the last survey on May 11, 2021. The only filter used was the publication date, with the previous 5 years having been selected.

The authors conducted research individually. Subsequently, the studies obtained were carefully analyzed by them and, in case of disagreement, a consensus was used to decide whether the article would be included in the review or not. As we retrieved the information needed for our study through reading the texts, no contact has been made with the authors of the ones selected.

The research on the PubMed/Medline database was conducted using the following describers: ( *chlamydia* [MeSH terms] OR *chlamydia* [all fields] OR *chlamydiae* [all fields] OR *chlamydias* [all fields]) AND ( *infertile* > [all fields] OR *infertilities* [all fields] OR *infertility* [MeSH terms] OR *infertility* [all fields] OR *infertile* [all fields] OR *infertility* [all fields]) AND (y_5 [filter]); *chlamydia* [MeSH terms] OR *chlamydia* [all fields] OR *chlamydiae* [all fields] OR *chlamydias* [all fields]) AND ( *tubal* [all Fields] AND ( *alter* [all fields] OR *altered* [all fields] OR *alteration* [all fields] OR *alterations* [all fields] OR *altered* [all fields] OR *altering* [all fields] OR *alters* [all fields]) AND ( *infertile* [all fields] OR *infertilities* [all fields] OR *infertility* [MeSH terms] OR *infertility* [all fields] OR *infertile* [all fields] OR *infertility* [all fields]); ( *chlamydia* [MeSH terms] OR *chlamydia* [all fields] OR *chlamydiae* [all fields] OR *chlamydias* [all fields]) AND ( *low* [all fields] AND ( *pregnancy* [MeSH terms] OR *pregnancy* [all fields] OR *pregnancies* [all fields] OR *pregnancy* [all fields]) AND *rates* [all fields]) AND (y_5 [filter]).

Firstly, in the identification phase, we found 325 studies through the database research and 2 by the references analyzed, leading us to 327 articles from which 5 were removed for being duplicates. Therefore, we had to screen 322 studies and, then, exclude 293 that failed to meet our eligibility criteria. So, in the eligibility phase, we assessed 29 articles. Finally, we removed seven studies for not having the possible correlation between CT infections and female infertility as the main focus, and three articles for being about STI in general. Moreover, two studies designed as reviews were ruled out too. Ergo, we included 17 studies in our qualitative synthesis.

## Results

**Fig. 1:**
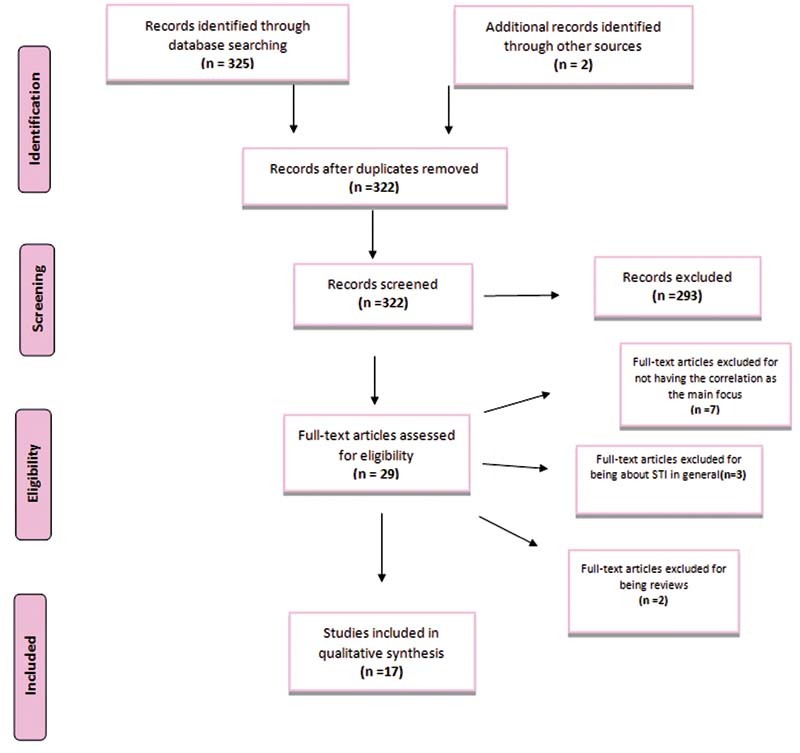
PRISMA flow diagram.

The flow diagram below ( Fig. 1 ), which is in line with the PRSIMA methodology ^9^ , shows that we screened 322 publications from the existing literature in the Medline/Pubmed database. Subsequently, 305 manuscripts had to be removed in view of the following criteria: The data presented was not focused on the correlation between infertility among reproductive-aged women and previous CT infection or on the comparison of fertility rates of infected and not infected women; were related to endocrine or male causes of infertility; were focused on STI in general or just in others different from CT infection; were designed as reviews. Hence, 17 studies were included in our systematic review.

The findings of our research are summarized in Chart 1 . As we can see, from the 17 manuscripts included, 8 were designed as cohort studies (including the regular, the retrospective and the longitudinal kind), 5 as case-control studies, 3 as cross-sectional studies and 1 as a letter- which was about a cross-sectional hospital-based study. In relation to the screening of CT infection, the methods used were very diversified- 4 used polymerase chain reaction (PCR), 2 enzyme linked immunosorbent assay (ELISA), 2 CT serology, 1 nucleic acid amplification test (NAAT), 1 major outer membrane protein (MOMP) and 1 automated DNA extraction method. Also, some works used more than one strategy- 1 CT serology and PCR, 1 MOMP and ELISA, 1 CT serology and NAATs and 1 CSI-PCR, CT serology and/or self-reported infection. In 2 articles, however, the method of screening was not specified. The association between CT infection and female infertility, which is our main focus, was considered positive in 13.

**Chart 1:** Results

Author (year)	Design	Journal	Methods of CT screening	Number of participants	Association between CT infection and female infertility	Main results
Menon et al. (2016) 10	Cross-sectional study	Journal of Medical Microbiology	ELISA	239	Positive	Up to half of women who are subfertile in this population could have CT as a cause or contributing factor.
Rawre et al. (2016) 11	Retrospective cohort study	APMIS	PCR	628	Positive	Significant association between rates of chlamydial infection and type of infertility, specially tubal factor infertility (74.7%; 56/75)
Davies et al. (2016) 12	Retrospective cohort study	The Lancet Infectious Diseases	Not specified	516,720	Positive	A positive CT test increased the risk of pelvic inflammatory disease, ectopic pregnancy, and tubal factor infertility by at least 30%.
Dehghan Marvast et al. (2017) 13	Case-control study	Andrologia	CT serology and PCR	324	Negative	In contrast to other studies, this study did not support the relationship between CT infection and TFI.
Ramadhani et al. (2017) 14	Letter	Sexually Transmitted Infections	PCR	290	Positive	CT was more highly associated with primary infertility
Zhu et al. (2017) 15	Case-control study	Reproductive Health	PCR	30,760	Positive	The prevalence of CT in subfertile couples in this study was 3.15% and increased yearly from 2.45% in 2010 to 3.69% in 2014.
Begum et al. (2017) 16	Cohort study	Mymensingh Medical Journal	ELISA	69	Positive	This study shows that by laparoscopy, significant number of cases of tubal and pelvic pathology was diagnosed in the chlamydia trachomatis seropositive subfertile female
Joolayi et al. (2017) 17	Case-control study	International Journal of Reproductive BioMedicine	MOMP and ELISA	225	Negative	6 (6%) infertile and 2 (1.6%) fertile women were positive for IgM ( *p* = 0.21). Also, PCR was positive for CT infection in 5 infertile (5%) and 2 fertile women (1.6%) ( *p* = 0.35). We did not find any seropositive immunoglobulin G in both groups.
Rantsi et al. (2018) 18	Cohort study	American Journal of Reproductive Immunology	MOMP	96	Negative	The overall pregnancy rate or live birth rate did not differ by the presence of antibodies or CMI against CT. Time to spontaneous pregnancy was longer among CT positive women
Kayiira et al. (2019) 19	Retrospective cohort study	Fertility Research and Practice	Are not specified	253	Positive	Exposure to current CT infection reduced chance of clinical pregnancy and a live birth after tubal flushing. Women with current CT infection had an increased risk of adverse events
Beyuo et al. (2019) 20	Cross-sectional study	International Journal of Gynecology and Obstetrics	CT Serology	189	Positive	CT infection was present in 7.9% of women with suspected TFI, which was confirmed in 35% of them.
Al-Farraj and Moubayed (2019) 21	Case-control study	Saudi Journal of Biological Sciences	Automated DNA extraction method	200	Positive	The percentage positivity to infection was significantly more among the infertile group com- pared to the control group.
Hoenderboom et al. (2019) 22	Cohort study	Sexually Transmitted Infections	PCR, CT serology and/or self-reported infection	13,498	Positive	This study adds to the evidence that chlamydia increases the risk for PID and TFI in women even if the infection was treated,29 but also showed that incidence rates were small.
den Heijer et al. (2019) 23	Retrospective cohort study	Clinical Infectious Diseases	CT serology and NAATs	857,324	Positive	Women who tested CT-positive had a substantially higher risk of experiencing female infertility (approximately 70%) than CT-negative women
Sukatendel et al. (2019) 24	Cross-sectional study	Open Access Macedonian Journal of Medical Science	PCR	50	Positive	The proportion of CT infection in tubal abnormality in this study was 66.7%, It was obtained that there was a significant relationship between CT infection with tubal abnormality (non-patency tubal) with *p* -value < 0.005 ( *p* = 0.001)
Hoenderboom et al. (2020) 25	Longitudinal cohort study	Sexually Transmitted Diseases	NAATs	5,704	Negative	Overall pregnancy rates were not lower in chlamydia-positive women compared with chlamydia-negative women, but among women with a pregnancy intention, time to pregnancy was longer and pregnancy rates were lower in chlamydia-positive women.
van Dooremalen et al. (2020) 26	Case-control study	Microorganisms	CT serology	891	Positive	CT antibodies were present significantly more often in the TFI+ compared to the TFI − group, respectively, 41.9% versus 9.6%

Abbreviations: CMI, cell-mediated immunity; CT, chlamydia trachomatis; DNA, deoxyribonucleic acid; ELISA, enzyme-linked immunosorbent assay; NAAT, nucleic acid amplification test; PCR, polymerase chain reaction; PID, pelvic inflammatory disease; TFI, tubal factor infertility.

## Discussion

This systematic review corroborates the hypothesis that CT infection potentiates female infertility, as 76.47% of the included studies found a positive correlation between them. The results of the study conducted by Menon et al., 10 which included 239 women, indicate that up to half of subfertile women could have CT as a cause or contributing factor. This is also expressed in den Heijer et al. 23 finding that CT-positive women had approximately 70% higher chance of experiencing infertility. Davies et al., 12 Ramadhani et al., 14 and Kayiira et al., 19 by presenting results that strengthen the discussed association, claim that policies of routine screening and interventions focused on preventing both first and repeated infections are extremely important in order to improve women's long-term reproductive health. 12 14 19

The type of female infertility more commonly associated with CT infection is tubal factor infertility (TFI), which occurs due to tubal occlusion (Toye et al., 1993). 27 According to Hoenderboom et al., 22 CT positivity represents a fourfold higher risk for TFI. In the study performed by van Dooremalen et al., 26 it is observed that CT antibodies were significantly more common among the group with TFI compared to the group without it, respectively 41.9% and 9.6%. 26 Additionally, Rawre et al. 11 supported this correlation, once they observed that 56 out of the 75 women with TFI had had CT infection. Nevertheless, the mechanism behind this interconnection is still unclear. 27

If we imagine PID with severe adherences and significant tubal damage, it is easy to conclude that an anatomic cause harms fertility. 28 29 Howbeit, there are some situations that do not visually present any alteration, which may suggest that there is also a molecular explanation. As CT is an intracellular pathogen that impairs the endothelium and the tubal muscle, probably it leads to an alteration in tubal motility and in endothelial cilia function. 30 This may explain the variations in intrauterine and tubal conformation, which presents areas of constrictions, which are observed during laparoscopy procedure, when a saline solution is inserted into the female reproductive tract. Even being fleeting there, CT facilitates the installation of other microorganisms in the female reproductive organs, causing a shift in its microbiota, with antigenic stimulus affecting the gametes and their conjunction. 31 32 33 This immunological alteration can also explain the mild endometriosis in patients that previously presented CT infection, once the immunological imbalance caused might lead to the impossibility of an adequate action of the lymphocytes, allowing the maintenance of viable endometrial cells in the pelvic environment. 34 Thus, the CT infection and its associated mechanical and biochemical damage, as well as endometriosis, induce a modification in the female reproductive tract's environment, which becomes hostile to the gametes.

Concerning the four articles that denied the association between CT infection and female infertility, Rantsi et al. 18 and Hoenderboom et al. 25 do affirm, however, that a longer time to conceive spontaneously was observed in women previously infected. This may indicate that past CT infection reduced the number of ciliated mucosal cells, leading to functional tubal damage and impairing the potential for pregnancy, even if it did not cause tubal occlusion. Joolayi et al. 17 point out some limitations of its study, such as the low number of the study population, the low number of women with secondary infertility, the short time of study, and the lack of real-time PCR.

We must mention that our review has two types of identified risks of bias—the publication and the selection bias. The first one is due to the fact that studies with a positive result have better chance of being published. Moreover, as we used only one database to find the articles, we might not have had access to articles on the subject published in other platforms, resulting in a selection bias. Also, there is a chance that we have not used all the proper keywords or that we failed to include in the review a useful study, which increases the last-mentioned type of bias.

## Conclusion

Even though a consensus among doctors about the matter is not established yet, this systematic review emphasizes that there is an important association between previous CT infection and female infertility, once the majority of publications analyzed confirms it. Evidence of tubal damage is highly suggestive of impaired fertility as a secondary consequence of this parasite infection, but there is a need for further studies on the possible molecular causes. Finally, we believe that making CT screening part of the infertility investigation routine is extremely relevant and has a reasonable justification.
